# Pathological or preventative? Amyloid-β as an effector of innate immunity

**DOI:** 10.1128/iai.00085-26

**Published:** 2026-06-26

**Authors:** Oluwagbenro O. Adesunloro, Amanda N. Tuckey, Jonathon P. Audia, Allyson E. Shea

**Affiliations:** 1 Department of Microbiology and Immunology, University of South Alabama Medical School 5557, Mobile, Alabama, USA; 2 Center for Lung Biology, University of South Alabama Medical School 5557, Mobile, Alabama, USA; 3 Department of Urology, University of South Alabama Medical School 5557, Mobile, Alabama, USA; University of California Merced, Merced, California, USA

**Keywords:** amyloid-β, infection, antimicrobial peptide, innate immunity

## Abstract

Amyloid-β (Aβ)-mediated inflammation, neurotoxicity, and plaque formation in the brain have long been regarded as pathological hallmarks of Alzheimer’s disease. However, emerging evidence indicates that Aβ also serves a normal physiological role in the innate immune defense against infectious agents. A growing body of literature supports the hypothesis that Aβ is produced in response to infection and is an antimicrobial peptide-like molecule capable of inhibiting microbial growth through mechanisms such as membrane disruption and microbial entrapment. Additionally, evidence positions Aβ as an innate immune effector that can induce pro-inflammatory signals during infection. In this minireview, we discuss the clinical, *in vivo*, and *in vitro* evidence supporting the antimicrobial and innate immune effector roles of Aβ in host defense. Collectively, these findings support a broader functional role for Aβ beyond its established association with Alzheimer’s disease pathology.

## INTRODUCTION

Alzheimer’s disease (AD) is a progressive neurodegenerative disorder primarily affecting the elderly and is characterized by a gradual decline in cognitive and brain function (1–4). Amyloid-β (Aβ) is established as a central component of the widely accepted amyloid cascade hypothesis of AD and a principal constituent of the hallmark plaques associated with neuropathology (5–7). Mechanistically, Aβ is thought to contribute to disease progression by driving synaptic dysfunction, processes that disrupt neuronal communication within the brain (8–10).

Aβ is generated from the sequential cleavage of amyloid precursor protein (APP) by β-secretases and γ-secretases (11, 12). This cleavage produces monomeric soluble Aβ peptides of varying lengths, with Aβ40 and Aβ42 being the most common isoforms (13–17). Once generated, Aβ monomers are amyloidogenic and spontaneously aggregate into oligomeric assemblies, including low-molecular-weight dimers and trimers, as well as larger protofibrils and insoluble fibrils (17). These fibrillar structures ultimately accumulate into large insoluble amyloid plaques, which are notably resistant to proteolytic degradation, detergents, heat, and can persist in tissue over time (18–21). Aβ was initially considered a pathogenic byproduct of APP metabolism with no specific cellular function; however, Aβ is now confirmed to be produced under normal physiological conditions and plays important roles in various cellular processes. Examples include maintenance of blood-brain barrier integrity, promotion of injury repair, regulation of synaptic function, and, most recently, antimicrobial defense (22–26).

One line of evidence supporting a physiological role for Aβ in innate immunity comes from experimental and structural analyses. Collectively, these studies indicate that Aβ shares key structural and functional features with antimicrobial peptides (AMPs), including the human cathelicidin LL-37 and several bacteriocins that kill or neutralize microbial pathogens (25–29). Like canonical AMPs, Aβ is an amphipathic, low-molecular-weight peptide lacking cysteine residues and possessing a hydrophobic C-terminus, a conserved heparin-binding motif, and an inherent propensity to oligomerize (30–32). Importantly, oligomerization and aggregation are recognized as physiological attributes of many AMPs that enhance antimicrobial efficacy (32). Together, these experimental and structural parallels positioned Aβ as a host defense peptide and provided a strong rationale for further experiments directly assessing its antimicrobial activity. This review explores the evolving view of Aβ as an immune effector molecule with antimicrobial properties.

## EVIDENCE FOR Aβ PRODUCTION IN HUMAN INFECTION AND ANIMAL MODELS

Aβ aggregation and plaque formation in the brain are well-established pathological features of AD (5). These observations raised the fundamental question of what environmental factor(s) trigger Aβ production in the brain to initiate disease onset. Early efforts to address this question focused on identifying stimuli capable of inducing rapid, localized Aβ expression in association with neuroinflammatory signaling. Infection emerged as a plausible candidate because microbial pathogens are known to access the central nervous system (CNS) and activate innate immune signaling. In this context, infection was not proposed as the cause of AD, but rather as a trigger of Aβ production that could become maladaptive over time (26, 33, 34).

Initial support for the infection hypothesis of AD came from reports of microbial pathogen co-localization with Aβ aggregates in post-mortem patient brain tissue. Identified remnants of infection included molecular and/or antigenic detection of Herpes simplex virus (HSV-1), *Chlamydia* (*Chlamydophila*) *pneumoniae*, spirochetes, human immunodeficiency virus, *Porphyromonas gingivalis*, as well as bacterial products such as lipopolysaccharide (LPS) (33, 35–42). The potential presence of these pathogens was associated with neuroinflammation and increased levels of inflammatory cytokines, APP, and Aβ (35, 37, 38, 41). Consistent with these post-mortem observations, Aβ42 has been detected at low but measurable levels in the cerebrospinal fluid of patients with active tuberculous meningitis, pneumococcal meningitis, and other forms of bacterial meningitis (43). This evidence suggests that Aβ accumulates within the human CNS during acute infection. Notably, the early studies exploring this possibility were met with substantial skepticism, in part due to their correlative nature and the prevailing view of amyloid aggregation as an exclusively pathological process.

Beyond associative human studies, support for infection as a potential upstream trigger of Aβ production has emerged from *in vitro* and *in vivo* model systems, including widely used AD-prone transgenic murine models that overproduce pathogenic Aβ (28, 44). Using transgenic 5×FAD mice and three-dimensional human neural cell culture infection models, Eimer et al. demonstrated that Aβ oligomers bind surface-associated glycoproteins on herpesviruses. Binding accelerates Aβ deposition, resulting in pathogen entrapment with reduced viral infectivity (28). Complementary *in vivo* evidence was provided by Kumar et al., who showed that 5×FAD mice challenged with *Salmonella enterica* serovar Typhimurium in a meningitis model exhibited significantly lower microbial burdens and improved survival compared to wild-type controls (44). Additionally, Aβ deposition was observed surrounding bacterial colonies, and intracerebral inoculation triggered rapid, localized Aβ accumulation at sites of infection, consistent with a classical innate immune containment response (44). Similar protective effects were observed in fungal infection models, where Aβ demonstrated antimicrobial activity against *Candida albicans* (44).

Further supporting a conserved role for Aβ in host defense across diverse classes of pathogens, bacterial antigens derived from *C. pneumoniae* and *P. gingivalis* have also been shown to induce the accumulation of Aβ in the brain (42, 45, 46). Likewise, challenge of mammalian glial and neuronal cells with *Borrelia* spirochetes led to increased Aβ deposition and APP expression (47). In addition, exposure to bacterial antigens such as lipopolysaccharide induced Aβ deposition in the hippocampus in a rat sepsis model (48). In contrast, mice genetically deficient in APP (and thus, Aβ production) displayed increased susceptibility to *S*. Typhimurium infection, signifying a protective role for APP and/or Aβ (44). Consistent with this interpretation, genetic ablation of β-secretase (BACE1), the rate-limiting enzyme for neuronal Aβ generation, is associated with increased mortality when mice are housed under non-specific pathogen-free conditions (49). This finding highlights the potential physiological cost of limiting Aβ production *in vivo*. Collectively, these studies support a model in which Aβ is induced in response to infections and functions as an innate immune effector that physically entraps pathogens and limits their dissemination.

Building on these foundational studies, additional clinical evidence indicates that infection-induced amyloid production is not restricted to the brain but also occurs at peripheral sites of infection, including the lung. One of the first reports came from studies of patients with severe pneumonia where amyloid species were detected in bronchoalveolar lavage fluid using conformation-dependent antibodies that recognize oligomeric amyloids, including Aβ (50). Subsequent investigations expanded these findings, demonstrating that pulmonary infection induces amyloidogenic proteinopathies within the lung endothelium and airspaces, with the potential to access the systemic circulation (51, 52). Several of these studies employed pan-amyloid detection strategies; therefore, not all data can be interpreted as Aβ specific.

Our recent work has shown that circulating Aβ levels are elevated in critically ill patients with systemic infection. In intensive care unit (ICU) cohorts, plasma Aβ levels were significantly increased in ICU patients with sepsis when compared to non-septic ICU controls or healthy individuals, indicating that Aβ induction is linked to infection-associated critical illness (53). In these patients, plasma Aβ40 levels correlated with disease severity, as assessed by Sequential Organ Failure Assessment scores. Furthermore, both Aβ40 and Aβ42 levels increased progressively from the time of ICU admission through 7 days of hospitalization. In addition, Aβ42 was detected in bronchoalveolar lavage fluid from patients with bacterial pneumonia using Aβ-specific assays (54). Together, these clinical studies support the concept that Aβ is induced during infection at both local and peripheral sites. These findings further reinforce the view that Aβ functions as a component of the innate immune response that may become dysregulated during severe or chronic infectious disease.

## PERIPHERAL SOURCES OF Aβ DURING INFECTION AND INFLAMMATION

APP is a type I transmembrane glycoprotein that is highly expressed in neurons, and Aβ is most widely recognized as a product of neuronal APP processing within the CNS (16, 55, 56). However, APP is also expressed in many cell types throughout the body, with substantial expression in peripheral tissues, including blood cells and endothelium (57, 58). The recent detection of Aβ in peripheral biological fluids, including ICU patient plasma (53) and bronchoalveolar lavage fluid (54), suggests that there are non-neuronal sources that may also contribute to Aβ production.

**Fig 1:**
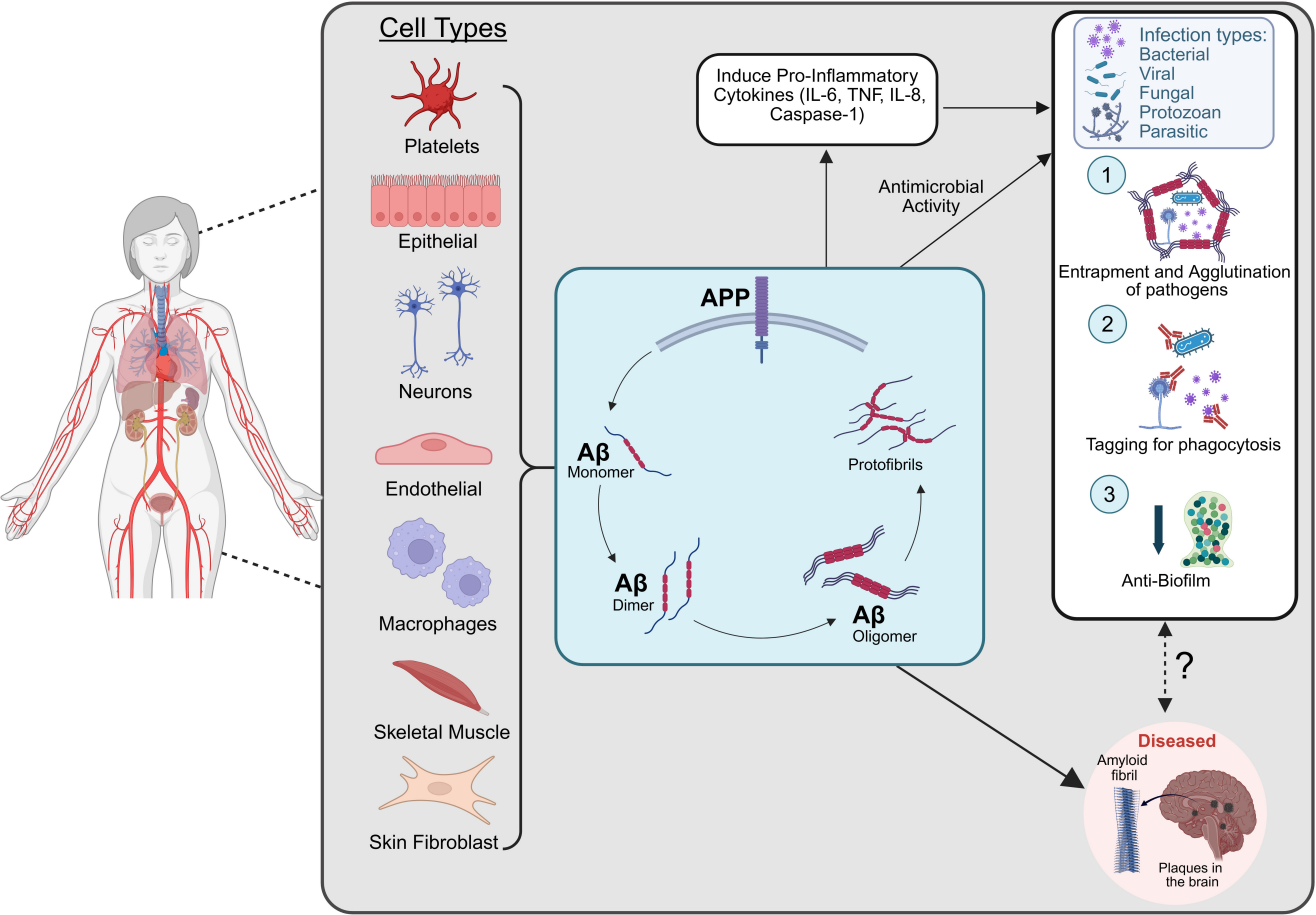
Peripheral expression of Aβ as an antimicrobial peptide and innate immune effector beyond the nervous system. Although APP and its cleavage product Aβ have been extensively studied in neurons, they are also expressed by multiple non-neuronal cell types, including platelets, macrophages, epithelial and endothelial cells, skeletal muscle cells, and fibroblasts. In these peripheral tissues, Aβ is predominantly produced in a monomeric form that can assemble into higher-order oligomers. These Aβ oligomers display potent antimicrobial activity, mediating direct pathogen entrapment and agglutination, promoting opsonization to enhance immune clearance, and disrupting microbial biofilms. This figure highlights the diverse cellular sources of Aβ and the dual functional roles of its aggregation states in host defense and disease pathology.

Membrane-associated APP can be proteolytically processed through two alternative pathways with distinct biological consequences (59). Sequential cleavage by β-secretases (BACE1 and BACE2) followed by the γ-secretase complex constitutes the amyloidogenic pathway, releasing Aβ peptides. In contrast, cleavage by α-secretase (ADAM 10/17) within the Aβ domain of APP generates a larger soluble fragment (sAPPα) and prevents Aβ generation (12, 60). Because APP undergoes complex and often rapid proteolytic processing, its full physiological functions remain incompletely understood. However, the presence of multiple biologically active domains within the full-length protein suggests roles extending beyond Aβ production, including cell adhesion and signaling (61). Furthermore, APP has been implicated in diverse physiological processes such as cell proliferation, neurite outgrowth, and synaptogenesis, underscoring its functional importance across tissues (60). Importantly, the balance between amyloidogenic and non-amyloidogenic APP processing appears to be cell-type specific and dynamically regulated, raising the possibility that inflammatory or infectious cues outside the brain may shift APP processing toward Aβ generation (62). As summarized in Fig. 1, multiple peripheral cell types express APP, Aβ, and the enzymatic machinery required for its processing, prompting the question of whether infection-driven Aβ production in the periphery is a conserved component of the host innate immune response.

### Platelets

Platelets represent the most abundant peripheral source of APP and Aβ (63–67). Platelets are small, anucleate cell fragments derived from megakaryocytes (platelet-forming cells) that circulate in the bloodstream and are classically known for their role in hemostasis, vascular repair, and immune regulation (68). Beyond coagulation, platelets actively participate in inflammation and host defense and are recognized as a major peripheral reservoir of Aβ. Platelets express abundant APP on their plasma membranes and within α-granules (64, 69). Upon activation of platelets, such as during clot formation, inflammation, or microbial encounter, platelets process APP via the β-secretase and γ-secretase pathways, leading to the release of Aβ peptides into the circulation (70). Platelet-derived Aβ can aggregate around pathogens, modulate inflammatory responses, and contribute to vascular immune defense, thereby linking hemostatic activity with antimicrobial and inflammatory pathways (71–73).

### Epithelial cells

Epithelial cells serve as a protective barrier between the internal environment and the external world, covering tissues such as the skin, respiratory tract, gastrointestinal tract, and the urinary tract (74). Beyond acting as a physical barrier, epithelial cells play crucial roles in secretion and absorption. They also contribute to immune defense by recognizing and responding to microbial threats through the expression of pattern recognition receptors (PRRs) and the release of antimicrobial peptides (75). Epithelial cells can also serve as a peripheral source of Aβ (76). APP has been found to be highly expressed in epidermis, a subset of epithelial cells, potentially leading to increased local release of Aβ through enhanced APP cleavage (77). Additionally, APP/Aβ has been found to be expressed in human colonic epithelial cell lines (Caco-2 cells). Specifically, these cells were found to secrete Aβ40 and Aβ42 under baseline conditions, which increase upon exposure to LPS (78). In addition, Aβ40 treatment of Caco-2 cells induced IL-6 release, highlighting the immunomodulatory role of Aβ in these cells (78). Likewise, APP is abundantly expressed in epidermal basal keratinocytes, melanocytes, and melanoma cells (77, 79). Within these cells, APP contributes to cell adhesion, migration, and proliferation through interactions with extracellular matrix components such as collagen, and sAPP serves as a growth factor that supports keratinocyte proliferation and wound repair (77, 79). Collectively, epithelial cells, particularly those of the skin and intestinal epithelium, constitute an additional peripheral source of APP and Aβ.

### Macrophages

APP is expressed in tissue-resident macrophages, including microglia and adipose tissue macrophages, and its expression is modulated by inflammatory signaling pathways (80, 81). In models of obesity-associated inflammation, APP has been implicated in macrophage activation within adipose and intestinal tissues, potentially linking APP expression to innate immune signaling. Pro-inflammatory stimulation of macrophages with TNF-α increases APP expression and is accompanied by elevated Aβ levels, further supporting a connection between macrophage activation and amyloid-mediated immunity (81). In addition, infection with *P. gingivalis* has been shown to promote Aβ accumulation in inflammatory monocytes and macrophages, suggesting that microbial challenge can enhance amyloidogenic processes within these cell types or enhance phagocytosis of extracellular Aβ (45). Collectively, these findings identify monocytes and macrophages as plausible peripheral sources of APP and Aβ during inflammatory or infectious states. However, evidence demonstrating Aβ production by macrophages *in vivo* remains limited, and additional studies are required to define the extent to which these cells contribute to circulating or tissue-associated Aβ during infection.

### Endothelial and other cells

The expression of APP, Aβ, and components of the amyloidogenic processing machinery extends beyond epithelial and immune cells to include multiple peripheral structural cell types. Recent work by Balczon et al. demonstrated that APP and the enzymatic components required for Aβ generation are present within the vasculature, implicating endothelial cells as potential contributors to APP/Aβ biology (82). In this study, APP was highly expressed in lung endothelial cells, alveolar epithelium, alveolar macrophages, and circulating monocytes. β-secretase (BACE1) was detected in capillary endothelial and alveolar epithelial cells at lower relative abundance, consistent with regulated rather than constitutive amyloidogenic processing (82). Beyond the lung and vasculature, APP expression has been documented in a wide range of peripheral cell types, including skin fibroblasts, skeletal muscle cells, osteoblasts, smooth muscle cells, and connective tissue-associated lineages (62, 83–87). The broad distribution of APP across vascular, muscular, and stromal tissues indicates that its expression is not restricted to specialized neuronal or immune compartments. Such ubiquitous expression may indicate that APP possesses context-dependent functions with the potential to impact host defense and tissue homeostasis.

## Aβ MECHANISMS OF IMMUNE ACTIVATION

Within the CNS, innate immune defense is mediated primarily by tissue-resident macrophages, including microglia, which function as key immune sentinels of the brain (88). Although the brain was historically described as an immune-privileged site, it is now recognized to mount robust but tightly regulated inflammatory responses to infection and tissue injury (89–91). In this context, Aβ has emerged as a functional component of innate immunity rather than solely a marker of neurodegeneration. Experimental evidence indicates that Aβ can act as an endogenous danger-associated signal capable of engaging innate immune sensing pathways (92). Consistent with this view, the induction of Aβ during infection may represent an innate host response within the CNS, although the extent to which this response is adaptive, context-dependent, or maladaptive remains an area of active investigation (93).

Aβ can engage innate immune signaling pathways through direct interaction with PRRs that normally detect microbial products or endogenous danger-associated molecules. Oligomeric and fibrillar assemblies of Aβ display structural features consistent with damage-associated molecular patterns and can be recognized by both surface-expressed and intracellular innate immune sensors (94). Among these, Toll-like receptors (TLRs) have been most consistently implicated. Studies in macrophages and microglia demonstrated that exposure to Aβ42 induces production of inflammatory cytokines such as TNF-α and IL-6, responses that are lost in cells deficient in TLR2 or TLR4 (95–97). These findings indicate that Aβ is recognized by innate immune cells using signaling pathways that overlap substantially with those used for microbial detection.

Innate immune activation by Aβ is not restricted to membrane-associated receptor signaling. Intracellular sensing pathways also contribute, particularly those involving inflammasome-forming nucleotide-binding oligomerization domain-like receptors (NLRs). Aggregated Aβ has been shown to activate NLRP3 and NLRP1 inflammasome pathways in microglia and/or macrophages, leading to caspase-1 activation and subsequent maturation of IL-1β from pro IL-1β (53, 98–100). More recent work links Aβ-induced NLRP3 inflammasome activation to alterations in cellular metabolism and mitochondrial function, consistent with engagement of pathways associated with cellular stress and damage (100). Together, these responses suggest that Aβ-driven inflammasome activation contributes to inflammatory signaling associated with chronic neuroinflammatory states.

Downstream of innate immune sensing, Aβ-driven signaling activates caspase-dependent pathways that amplify inflammatory responses. Caspase-1 activation promotes maturation and release of IL-1β and IL-18 and can induce pyroptotic cell death, while noncanonical inflammasome-associated caspases (caspase-4/5 in humans, caspase-11 in mice) further contribute to inflammatory amplification and cell injury (100). Through these pathways, Aβ directly couples innate immune sensing to defined effector mechanisms that shape tissue responses during infection and injury. These observations support a model in which Aβ functions as an endogenous immune-activating ligand that engages canonical host defense pathways. Notably, the same structural and biochemical properties that drive this immune activation also confer the capacity to bind and neutralize microbial pathogens.

## ANTIMICROBIAL ACTIVITY OF Aβ

A compelling body of experimental evidence supports the idea that Aβ functions as an antimicrobial molecule (25, 101, 102). This includes a combination of *in vitro* assays and *in vivo* infection models that demonstrate the capacity of Aβ to inhibit microbial pathogens. Two primary mechanisms have been proposed for Aβ-mediated microbial clearance. First, Aβ can act as an anti-adhesive molecule, coating pathogens to prevent their attachment to host cells and inhibit their growth. Second, Aβ functions as an opsonin, marking microorganisms for enhanced recognition and uptake by phagocytic cells (103). These two mechanisms are further described below.

### Antibacterial effect of Aβ

Multiple studies have demonstrated that Aβ exerts bactericidal and bacteriostatic activity against a broad panel of gram-positive and gram-negative bacteria (25). A foundational study by Soscia et al. demonstrated that Aβ possesses broad-spectrum antimicrobial properties. Synthetic Aβ peptides, including Aβ40 and Aβ42, were tested against a range of microorganisms, including *Escherichia coli*, *Staphylococcus aureus*, and *Streptococcus pneumoniae*, with efficacy comparable to the human antimicrobial peptide LL-37 (25). Mechanistic analyses indicate that Aβ can disrupt microbial membranes, although this activity appears to occur in conjunction with additional mechanisms. Notably, Aβ contains structural features that enable binding to glycosaminoglycans and other microbial surface glycans, which may facilitate initial interactions with bacterial cell surfaces (81). In addition to membrane disruption, Aβ has been shown to agglutinate microbes. Electron microscopy revealed that Aβ fibrils bind to and entrap microbial cells, forming dense aggregates that likely prevent pathogen spread (103) and enhance engulfment by phagocytes (101, 102). These effects are consistent with the ability of Aβ to form cross-β fibrillar networks that act as a scaffold for pathogen sequestration (103). Moreover, Aβ can form fibrillar meshes that trap and neutralize pathogens, preventing their dissemination (103), a function that is reminiscent of entrapment mechanisms observed in neutrophil extracellular traps (NETs) (104). Consistent with these observations, Aβ overexpression in transgenic model-derived cell cultures, or treatment with synthetic Aβ, reduces bacterial burden (44). Together, these findings support a model in which Aβ exerts antimicrobial activity through a combination of membrane perturbation, surface binding, and fibril-mediated pathogen sequestration.

Recent work has identified a direct interaction between bacteria-derived amyloids and host Aβ, revealing a mechanism by which microbial products can modulate Aβ oligomerization (101, 102). Using co-culture models of neuroblastoma cells and uropathogenic *Escherichia coli* (UPEC), Prosswimmer et al. show that bacterial amyloid production, specifically curli fibers composed of the CsgA protein, induces the formation of Aβ oligomers. These oligomers disrupt bacterial amyloidogenesis, leading to reduced curli production and decreased biofilm biomass. As a result, UPEC becomes more susceptible to gentamicin treatment and is more efficiently cleared by macrophages (102). Similar findings were reported by Syed et al., who demonstrated that cerebrally produced Aβ can enter the systemic circulation and selectively co-localize with microbial amyloids in peripheral tissues (105). Complementary *in vitro* assays revealed that Aβ monomers selectively recognize and disassemble bacterial amyloids FapC and CsgA, derived from *Pseudomonas aeruginosa* and *E. coli*, respectively. This disassembly compromises biofilm structure, impairs bacterial adhesion to intestinal epithelial cells, and increases sensitivity to antibiotics (105).

### Antiviral effect of Aβ

Aβ has also been shown to neutralize viruses. White et al. demonstrated that Aβ exhibits potent antiviral activity against multiple strains of Influenza A virus, including seasonal and pandemic H1N1 and H3N2 variants (106). The antiviral effect was dose dependent and more pronounced in Aβ42 than in Aβ40 or scrambled peptides. Direct interaction between Aβ42 and the virus was essential, as pre-incubation with Aβ42 reduced viral uptake by epithelial cells and induced viral aggregation, without causing cytotoxicity. Beyond neutralization, Aβ42 enhanced neutrophil responses, including hydrogen peroxide production and NET formation. This led to increased phagocytic uptake of influenza A virus by neutrophils and monocytes. Although Aβ42 promoted monocyte uptake of the virus, it suppressed viral protein synthesis and reduced IL-6 production without affecting TNF-α, indicating a selective immunomodulatory effect (106). Similarly, Bourgade et al. provide compelling evidence that Aβ40 and Aβ42 have antiviral activity against HSV-1. In human fibroblast, epithelial, and neuronal cell lines, Aβ significantly inhibited HSV-1 replication in a sequence-specific manner when present prior to or during infection, but not afterward, suggesting that Aβ effect is limited to early stages of viral entry (107). Mechanistic studies demonstrated that Aβ acts extracellularly by directly binding HSV-1 virions to prevent attachment or fusion with host membranes. These antiviral properties parallel those of classical AMPs like LL-37, though with distinct temporal dynamics (107). These findings support the classification of Aβ as a functional antimicrobial peptide with both direct antiviral activity and the ability to modulate host immune responses.

### Antifungal effect of Aβ

In addition to studies examining the antibacterial and antiviral activities of Aβ, Kumar et al. demonstrated that transformed cells overexpressing Aβ are protected against *Candida albicans* (44). This protection occurs through disruption of fungal adhesion to host cells and promotion of microbial agglutination, suggesting an antifungal role for Aβ. These effects are mediated by oligomeric Aβ interacting with fungal cell wall carbohydrates. However, non-transformed cells lacking the ability to secrete Aβ abolished these activities. These findings indicate that Aβ restricts fungal colonization, highlighting its potential role in innate immune defense (44).

### Potential antimicrobial activity of Aβ: perspectives and future directions

The presence of Aβ in the systemic circulation, including at basal levels in healthy individuals (53, 108), raises the possibility that Aβ may contribute to host defense beyond the CNS. Peripheral Aβ is generated in part by platelets and other non-neuronal sources, as discussed earlier. Once released into circulation, Aβ peptides can bind and aggregate through interactions with sulfated glycans, including heparan sulfate proteoglycans, glycosaminoglycans, and gangliosides, or engage multiligand scavenger/lipoprotein receptors (109–112). Interestingly, these same classes of molecules are also utilized by many viruses, such as SARS-CoV-2 and herpes simplex virus, to concentrate at the cell surface prior to entry (113–115). In this context, Aβ may exhibit competitive or inhibitory interactions with disseminating pathogens. At the cell surface, Aβ and viral particles may compete for access to glycan-rich receptors. Alternatively, because Aβ uses these surfaces as nucleation templates for oligomerization, such assemblies could promote virion agglutination, opsonization, or physical entrapment, thereby inhibiting viral entry. These proposed indirect antiviral mechanisms remain highly speculative. Nonetheless, they suggest a potential mechanistic link between circulating Aβ, pathogen surface recognition, and the restriction of viral systemic spread.

Extending this concept beyond viral pathogens, it is conceivable that Aβ may also interact with glycoproteins involved in bacterial and fungal adherence. Depending on the systemic distribution and localized production, these interactions may also be relevant in acute infection contexts. For example, many uropathogens utilize mannosylated residues on urinary tract epithelial cells for attachment and entry (116). However, additional mechanistic and *in vivo* studies are required to determine whether such interactions are biologically meaningful.

## FAILED IMMUNE RESOLUTION AND THE LONG-TERM CONSEQUENCES OF Aβ INDUCTION

An important issue raised by the studies reviewed here is whether pathological Aβ accumulation reflects incomplete immune resolution following repeated infectious or inflammatory challenges. Across experimental and clinical contexts, Aβ is consistently observed to be induced during innate immune activation in response to infection and tissue stress. Multiple central and peripheral cell types are capable of producing Aβ, which adopts assembly states that support antimicrobial activity, pathogen containment, and immune signaling. Under physiological conditions, these responses are expected to be transient and tightly regulated. However, when immune resolution is impaired, sustained Aβ production may persist and contribute to chronic inflammation. Thus, Aβ accumulation may represent a molecular imprint of unresolved innate immune activation, consistent with the AD2 paradigm, which emphasizes chronic engagement of innate immune pathways as a driver of Alzheimer’s disease pathogenesis (32).

Related insights emerge from post-infectious clinical syndromes characterized by prolonged immune dysregulation. Post-intensive care syndrome (PICS) describes patients who survive critical illness yet exhibit persistent inflammation, immune dysfunction, metabolic abnormalities, and cognitive impairment (117). Similarly, post-acute sequelae of SARS-CoV-2 infection (PASC) are associated with sustained inflammatory signaling and cognitive symptoms long after viral clearance, particularly in older adults (118). In both conditions, neurologic sequelae arise in the absence of ongoing infection and instead reflect a failure to appropriately terminate innate immune programs. These observations suggest that prolonged immune activation can exert lasting effects on brain function, shaped by cumulative immune challenges over time.

Clinical studies in critically ill patients provide further insight into the systemic dimensions of Aβ biology. In sepsis, circulating Aβ levels are markedly elevated and correlate with disease severity and organ injury (53). The kidney is a major site of peptide clearance, and acute kidney injury is common during sepsis, suggesting that renal dysfunction may contribute to sustained elevations of circulating Aβ (53, 119). At the same time, prolonged exposure to circulating Aβ may itself influence distal organ function, creating reciprocal interactions between immune activation, impaired clearance, and tissue injury. These findings link systemic infection, organ dysfunction, and altered Aβ dynamics and provide a mechanistic context for how peripheral immune events may influence CNS pathology.

A remaining question is whether Aβ induction is restricted to severe systemic infections or represents a more general feature of host responses to microbial challenge. While current evidence is strongest in sepsis and meningitis, it remains unclear whether localized or acute infections also elicit measurable Aβ production. Ongoing studies in pneumonia and urinary tract infection will be important for determining whether Aβ induction scales with infection severity or occurs broadly across infectious contexts. Resolving this question will clarify whether Aβ functions primarily as an emergency response molecule or as a more routinely deployed component of innate immunity.

Taken together, these observations support a view of Alzheimer’s disease as a process that may unfold over decades, shaped by cumulative infectious and inflammatory exposures. In this model, each immune challenge may induce transient Aβ production. When immune resolution is successful, Aβ levels normalize. When resolution fails, Aβ persists and may ultimately contribute to sustained neuroinflammation. Framing Alzheimer’s disease through the lens of failed immune resolution, alongside PICS and PASC, highlights the importance of understanding not only how innate immune responses are initiated, but how they are terminated.
